# Current Practice in Pediatric Cow’s Milk Protein Allergy–Immunological Features and Beyond

**DOI:** 10.3390/ijms24055025

**Published:** 2023-03-06

**Authors:** Vanessza Emmert, Dominika Lendvai-Emmert, Kata Eklics, Viktória Prémusz, Gergely Péter Tóth

**Affiliations:** 1 Doctoral School of Health Sciences, Faculty of Health Sciences, University of Pécs, 7621 Pécs, Hungary; 2 Erzsébet Teaching Hospital and Rehabilitation Institute, 9400 Sopron, Hungary; 3 Department of Neurosurgery, Medical School, University of Pécs, 7623 Pécs, Hungary; 4 Neurotrauma Research Group, Szentágothai Research Centre, University of Pécs, 7624 Pécs, Hungary; 5 Department of Languages for Biomedical Purposes, Medical School, University of Pécs, 7624 Pécs, Hungary; 6 Institute of Physiotherapy and Sport Sciences, Faculty of Health Sciences, University of Pécs, 7621 Pécs, Hungary

**Keywords:** cow’s milk protein allergy, food allergy, IgE-mediated, non-IgE-mediated, epitope, immunotherapy

## Abstract

Cow’s milk protein allergy is one of the most common pediatric food allergies. It poses a significant socioeconomic burden in industrialized countries and has a profound effect on the quality of life of affected individuals and their families. Diverse immunologic pathways can lead to the clinical symptoms of cow’s milk protein allergy; some of the pathomechanisms are known in detail, but others need further elucidation. A comprehensive understanding of the development of food allergies and the features of oral tolerance could have the potential to unlock more precise diagnostic tools and novel therapeutic approaches for patients with cow’s milk protein allergy.

## 1. Introduction

Cow’s milk protein allergy (CMPA) poses a constant challenge for healthcare providers and patients alike. The increasing incidence of food allergies in industrialized, urban environments, the diagnostic difficulties physicians may face, the cost associated with the morbidity [1], and the significant influence on the quality of life for affected individuals make cow’s milk protein allergy research important and needed to this day.

Cow’s milk allergy is a reproducible hypersensitivity reaction, consistently leading to a wide variety of symptoms, including gastrointestinal, respiratory, and skin manifestations, after the ingestion of the culprit food.

The prevalence of the condition among infants is 2–6% and decreases with age, although the onset of symptoms may occur at any age [2,3]. Approximately 60% of patients have IgE-mediated CMPA. The exact prevalence is difficult to monitor since a significant proportion of studies relies on self-reported symptoms, and the number of studies involving only clinically proven CMPA is scarce [4,5]. Based on national population estimates (mainly from the United States) regarding IgE-mediated cow’s milk protein allergy, a peak prevalence in young children between 1–5 years of age is observed (approximately 20% sensitized and an estimated clinical food allergy rate of approximately 2%). Prevalence shows a decreasing tendency with age. In older children and adult population, prevalence is estimated to be around 0.16% to 0.49% [6]. 

The epidemiology of non-IgE-mediated CMPA has been subject to fewer studies. Some national cohorts are helpful in estimating the prevalence of different conditions. FPIAP, as the most common disorder with non-IgE-mediated background, has been associated with a wide prevalence range of 0.16 to 7%, and the cumulative incidence is described to be around 17%. FPE shows a progressively decreasing incidence over time, and some studies indicate a prevalence rate of around 2%. FPIES is considered to be a rare condition; the cumulative incidence is estimated to be 0.3–0.7% in infancy [6]. The prognosis of CMPA is mostly favorable, and the majority of cases resolve by the school age [6,7].

The aim of this review is to summarize the immunologic background of CMPA, the mechanism of oral tolerance, and possible treatment strategies already in use or under investigation.

## 2. Classification and Clinical Symptoms of CMPA

The two main subtypes of CMPA, based on the immunologic response to the allergen and the subsequent occurrence of symptoms, are IgE- and non-IgE-mediated allergies, although a mixed presentation caused by activation of both immunologic pathways also exists [7,8] (Table 1).

Patients with IgE-mediated allergy show symptoms within minutes up to 2 h of culprit food ingestion; the severity ranges from relatively mild manifestations to life-threatening anaphylaxis, whereas non-IgE-mediated patients’ symptoms develop after a delay, usually within hours or days, rarely weeks.

IgE-mediated CMPA can affect many different organ systems, mostly the skin and the gastrointestinal and respiratory tracts; the most frequently observed symptoms associated with this condition are acute urticaria, angioedema, pruritus, oral tingling, oral pruritus, swelling, nausea and/or vomiting, abdominal pain, wheezing, and systemic symptoms, such as hypotension, hypothermia, and potentially fatal anaphylaxis [9,10].

The clinical manifestations of non-IgE-mediated CMPA can be described as three clinical disorders: food protein-induced enterocolitis syndrome (FPIES), food protein-induced enteropathy (FPE), and food protein-induced allergic proctocolitis (FPIAP); an overlap between clinical symptoms is common. The severity of these manifestations represents a spectrum, with FPIAP being the most benign and FPIES usually being the most severe [7].

The leading symptom of FPIES is profuse and repetitive vomiting, which can be followed by diarrhea in up to half of the patients. Lethargy, pallor, and hypothermia may also occur; rarely, hemodynamic instability develops in the affected patients [11].

The clinical presentation of FPE is similar to celiac disease: after the introduction of cow’s milk to the infant’s diet, chronic diarrhea and malabsorption with indicative signs, such as steatorrhea and failure to thrive, develop. In contrast to FPIES and FPIAP, FPE may require endoscopy and histologic analysis to confirm the diagnosis [12].

FPIAP usually develops within the first weeks of life of breastfed infants; they are exposed to cow’s milk protein through the maternal intake. Loose, bloody stools, in some instances with mucus, show inflammatory changes of the rectum and colon in otherwise healthy-appearing infants [7,12].

Eosinophilic gastrointestinal disorders (eosinophilic esophagitis, gastritis, gastroenteritis, and colitis) represent a distinctive group of illnesses among cow’s milk protein allergy manifestations. The exact pathophysiology of eosinophilic gastrointestinal disorders (EGIDs) is still under investigation, but they are typically categorized as “mixed” IgE and non-IgE mediated because allergic sensitization is often observed [12]. Clinical presentation includes vomiting, heartburn, regurgitation, and abdominal pain [13]. Eosinophilic disorders are closely related to atopy: a high proportion of patients (about 70%) present with a history of atopy [14].

## 3. Allergen Composition of Cow’s Milk

The total protein composition of cow’s milk can be divided into two main fractions, which are obtained through the acidification of raw milk (pH 4.6 at 20 °C): the coagulum containing casein proteins (80% of total milk proteins) and the lactoserum (whey proteins, 20% of total milk proteins). The casein fraction contains αs1-, αs2-, β-, and κ-caseins and three γ-caseins derived from the hydrolysis of β-casein (the latter being mostly abundant in cheeses rather than milk). Caseins are resistant to heat but susceptible to enzymatic degradation. One of the most important components of lactoserum is β-lactoglobulin, which contains linear IgE binding epitopes. Other components include α-lactalbumin (which, despite a high amino acid sequence similarity to its human counterpart, contains genuine, milk-specific epitopes) and bovine serum albumin (which presumably plays a role not only in CMPA but also in beef allergy); finally, the allergenic activity and clinical significance of some components, such as immunoglobulin G and lactoferrin, require further investigation [15,16] The main characteristics of the mentioned allergens is summarized in Table 2.

Patients with IgE antibodies against casein were found to have a higher likelihood of allergy persistence [17].

## 4. Effect of Technological Methods in Cow’s Milk Processing on Allergenicity

Heating is a fundamental step for enabling the commercial availability of milk because of the reduction or elimination of microorganisms; during pasteurization, temperatures below boiling point are applied, whereas ultra-high-temperature processing uses temperatures exceeding 100 °C. Powdered, milk-based infant formula is produced through evaporation, during which exposure to high temperatures is usually short. However, heating seems to have a moderate effect on the allergenic potential of milk proteins, though some authors found a reduction in whey protein allergenicity. High temperatures can only modify conformational epitopes, whereas linear epitopes remain unaffected; hence they are still able to maintain their allergenic potential.

Hydrolysis is utilized to alter the allergenicity of milk proteins, but only extensively hydrolyzed formulas are considered safe for patients with CMPA (in contrast to partially hydrolyzed products). A combination of ultrafiltration (which removes any undigested proteins remaining in the filtrate after proteolysis) and hydrolysis can further improve the safety of hypoallergenic products [16].

Although some patients are able to tolerate baked milk, as a higher number of Treg cells and lower milk-specific IgE levels were detected in this patient population [14,18,19], another factor possibly associated with the reduced allergenicity of baked milk products is the interaction between milk proteins and some components of the food matrix (e.g., wheat); milk allergens embedded in a matrix showed consistently reduced allergenicity compared to heated milk [20].

## 5. Pathophysiology of CMPA and the Background of Oral Tolerance Formation

### 5.1. IgE Mediated CMPA

The key feature in food allergy pathogenesis is the lack of oral tolerance–a normal state of unresponsiveness–to food allergens. 

Sensitization is the preceding step in the development of symptomatic food allergy, and the “dual allergen exposure hypothesis” sheds light on multiple important mechanisms leading up to the development of food allergy. As opposed to the previous theory about sensitization, which emphasized the role of intestinal exposure (via breast milk or oral food ingestion) to dietary antigens, increasing evidence indicates an important role of early life allergen exposure through the skin in the subsequent manifestation of food allergy, whereas early oral exposure can establish tolerance [21,22]. The exact mechanisms by which a Th2-mediated response of cutaneous origin can reach the gut remain unclear, although (based on animal models) it has been proposed that dendritic cells (DCs) in the skin may trigger allergen-specific T cells through retinoic acid production to express gut-homing markers [23,24].

Food antigens are digested into peptides and amino acids; however, a small fraction can reach the intestinal epithelium in an intact form, which enables interaction with antigen-presenting cells.

Dietary antigens are able to pass through the intestinal epithelium, depending on size and solubility, to subsequently access antigen-presenting cells in the mucosa via passive or active transport. Passive transport, also known as paracellular diffusion, happens when a food antigen passes between two adjoining enterocytes.

Under homeostatic conditions, tight junctions between enterocytes prevent the paracellular passing of antigens. Increased transport of intact antigen through epithelial cells has been related to its allergenic activity.

The active system takes place through different cell types. Microfold (M) cells are flattened epithelial cells overlying Peyer’s patches; sampling through these cells is associated with the induction of IgA production, which plays a critical role in the intestinal lumen. Goblet cells also take part in soluble antigen transport by creating goblet-cell-associated antigen passages (GAPs), which then deliver antigens to CD103+ CX3CR1- dendritic cells, a DC subset associated with the development of tolerance. Mucin secretion increases antigen transport through goblet cells and uptake by the aforementioned DC subset [25]; additionally, hyperglycosylated mucin MUC2 has a conditioning effect toward a regulatory phenotype for CD103+ DCs and intestinal epithelial cells [26].

Specialized macrophages with CX3C receptor 1 expression are able to pass captured antigen via trans-epithelial dendrites or phagocytosis onto DC for transport to mesenteric lymph nodes (MLNs) to prime immune responders, such as lamina propria DCs [27]. CD103+ dendritic cells in the lamina propria are capable of promoting differentiation of naïve T cells into antigen-specific forkhead box P3 (FOXP3+) regulatory T (Treg) cells and possibly, into FOXP3-, IL-10-secreting, type 1 regulatory T cells [24,26,28,29].

TGFß-expressing Th3 cells are induced after ingestion of antigen which shows some overlapping functions and possible plasticity with Foxp3+ T cells. Tr1 cells have immunosuppressive effects through IL-10 and, thus, are capable of preventing colitis, although their exact role in oral tolerance could not yet be determined because of conflicting research results [26].

The transport of antigens to secondary lymphoid tissues, such as mesenteric lymph nodes or Peyer’s patches, promotes tolerance, although the exact importance of these secondary lymphoid organs needs further investigation [30,31,32].

A very small fraction of proteins (an estimated 2%) passes through the intestinal epithelium in intact form, which then could be transported to the liver or secondary lymphoid tissues for antigen presentation [25]. After a high-dose antigen exposure, through a mechanism mediated by plasmacytoid DCs in the liver and mesenteric lymph nodes, the deletion of antigen-specific CD8+ and CD4+ T cells induces oral tolerance [33,34].

Multiple Treg populations play a role in the development of oral tolerance, especially antigen-specific CD4 + CD25 + Foxp3 + Tregs, which is the most widely studied and understood group of Treg subsets, with a crucial part in the intestinal tolerance [35].

Another factor in the complex development of oral tolerance is homing of Treg cells to the gut, which is promoted by the production of retinoic acid by CD103+ DCs and the subsequent expression of integrin α4ß7. A further human study that underlines the quintessential role of Treg cells showed that hypomethylation of the Foxp3 locus leads to its increased accessibility for transcription in patients who respond favorably to oral immunotherapy (development and maintenance of functional tolerance) [24].

A potential capacity of tonsils in the development of oral tolerance has been described. The anatomical location and abundance of CD4+ Foxp3+ Tregs and allergen-specific Tregs indicate that these organs may represent the first phase of oral tolerance induction to food allergens [36].

Besides the induction of regulatory Treg cells, anergy (unresponsiveness to the antigen) and T-cell depletion (apoptosis of antigen-specific T cells) are also mechanisms through which oral tolerance can be achieved. The former phenomenon occurs after high-dose antigen exposure, while the latter follows low-dose exposure [26].

In patients with IgE-mediated food CMPA, the physiologic tolerance is disrupted, for example, after exposure to pathogen-associated molecular patterns (PAMPs) or epithelial damage, which leads to IL-25, IL-33, and thymic stromal lymphopoietin (TSLP) production. These changes cause an alteration in the induction of Treg cells, which are subsequently switched to antigen-specific Th2 cells. Th2 cells stimulate B cells through IL-4 production, which then results in immunoglobulin E (IgE) production and mast cell expansion. Tolerogenic Treg function is suppressed by IL-4, and finally, these cells are reprogrammed to produce IL-4 themselves, transforming from tolerogenic to pathogenic ones [37].

Type 2 innate lymphoid cells (Th2-like cells without antigenic specificity), which secrete IL-4 and IL-13, further inhibit Treg activity [29]. Secreted IgE binds the surfaces of basophils and mast cells. If the patient is repeatedly exposed to the food antigen, it binds food-specific IgE attached to Fcε receptors on mast cell and basophil cell surfaces, which leads to degranulation of the previously mentioned innate immune cells. The release of allergic mediators, such as histamine, is responsible for the development of immediate allergic reactions [38].

### 5.2. Non-IgE Mediated CMPA

The molecular background of non-IgE-mediated CMPA is understood in far less detail than the IgE-mediated variant. Cellular immunity is presumed to be the key factor in the allergic response in the absence of circulating sIgE, although localized intestinal IgE response has been previously described [39].

Research indicates the presence of food-specific T cells in FPIES, but their distinctive role is yet to be identified. Although Th2 responses are usually described in association with IgE-mediated allergy, their apparent role in FPIES (with increased production of IL-4, IL-5, IL-9, and IL-13) can be explained by the high rate of atopy as a comorbidity in these patients [40]. Studies have shown a broad activation of the innate immune system (monocytes, neutrophils, eosinophils, and natural killer cells) in patients with FPIES after an oral food challenge. Mast cells also seem to play a role in the development of allergic symptoms, as indicated by the in vitro production of IL-9 and significantly higher baseline levels of tryptase in FPIES patients with a positive oral food challenge (OFC). A higher IL-8 level in this patient group is associated with neutrophil involvement [7,41].

Structural damage to the jejunal mucosa and subsequent malabsorption are important features of FPE, which seem to be caused by food-specific T cells (predominantly cytotoxic CD8 + T cells) infiltration [7,42].

Dense eosinophilic infiltration of the rectosigmoid mucosa is characteristic of FPIAP. Since breastfed infants are predominantly affected by the disease, a potential role of immunologic components in breast milk and their interaction with dietary proteins has been proposed [7].

Eosinophil gastrointestinal disorders are characterized by abnormal eosinophilic infiltration of the esophagus, stomach, small intestine, or colon, leading to organ dysfunction and clinical symptoms. Although the pathomechanism of these conditions still needs further clarification (eosinophilic esophagitis is the most studied subtype of these disorders), available data indicates an allergic background (caused by food allergens such as cow’s milk, peanut, or egg) of mixed immunologic mechanisms (IgE- and non-IgE-mediated) [13].

The central role of the Th2 response in the pathogenesis of eosinophilic esophagitis, with the release of mediators such as thymic stromal lymphopoietin (TSLP), IL-4, IL-5, IL-13, TGFß, and eosinophil chemokines (eotaxin 1-3/CCL11-CCL24-CCL26 and RANTES/CCL5) has been demonstrated [43]. A lower peripheral and a higher esophageal level of invariant natural killer T cells (iNKTs)–a subset of T cells activated by sphingolipids in cow’s milk, usually associated with IgE-mediated food allergy–has been described in patients with eosinophilic oesophagitis [14,44].

CMPA has been linked to a variety of nonspecific symptoms, such as allergic dysmotility (GERD, dyspepsia, and constipation), without a complete understanding of the underlying pathomechanisms. Animal models indicate a significant modulating role of allergic responses on intestinal motility. A Th2-dominant response to food allergens causes the release of IL-4 and IL-13, which alter smooth muscle motility and spontaneous contractility via the TGFß upregulation [45]. Another animal model indicated that tissue infiltration of IgE-degranulating mast cells in the mucosa and mesenteric lymph nodes causes loose stools and poor weight gain [46]. Mast cells are capable of generating sensorimotor dysfunction of the gut and play a role in functional dyspepsia in atopic patients [14,47].

## 6. Diagnosis: Current Practice and Emerging Options

The initial steps toward the diagnosis of CMPA are a thorough clinical history (preferably guided by open-ended questions) and a physical examination. If an IgE-mediated allergy is suspected, measurement of specific serum IgE levels (sIgE) and a skin prick test (SPT) are a reasonable next step, though it should be noted that these tests alone are insufficient for establishing the diagnosis of CMPA [9,48]. 

SPT has a high negative predictive value, but a positive test is not suitable for confirming the diagnosis of CMPA; it rather indicates a state of sensitization. An allergen extract is transferred to the tip of a small lancet, which penetrates the epithelial barrier, causing mast cell degranulation in susceptible individuals, which presents as a wheal-and-flare reaction [24].

The main advantage of measuring specific serum IgE levels is the correlation of s-IgE concentrations with the possibility and severity of a clinical reaction to the allergen, although an exact cutoff value is difficult to determine (levels vary with age and the type of allergen) [29].

Before the subsequent OFC, which is still the gold standard for establishing the diagnosis of food allergy, the aforementioned diagnostic methods are appropriate for risk assessment.

During OFC, increasing doses of the suspected allergen are administered orally until either clinical symptoms develop or a maximum tolerated dose is reached [24].

It is ideal for conducting it in a double-blind, placebo-controlled (DBPCFC) design, but due to the high costs and time commitment required, it is rarely used in the clinical setting. In addition, oral food challenges should take place in specialized hospital wards with personnel and equipment suitable for promptly managing acute allergic reactions.

Fecal calprotectin has been successfully utilized for the diagnosis and monitoring of gastrointestinal inflammation over the last decades because the quantification of this biomarker is a simple, fast, and relatively inexpensive procedure. It may be a valuable tool, as some studies have recently demonstrated, in the medical diagnosis of the non-IgE-mediated CMPA [2].

A promising direction toward a more precise and objective diagnosis of food allergies is component-resolved allergy testing. In this method, purified or recombinant allergens are used to identify allergen-specific IgE and IgG4 antibodies [49]. A significant advantage of this method in the pediatric population is the smaller amount of blood serum needed to perform the test compared to conventional assays; even capillary blood sampling can be performed. However, the efficacy of this diagnostic tool and its possible superiority over conventional s-IgE measurement and SPT need further evaluation due to conflicting reports from previous studies. A notable area in which component-resolved diagnostics could be beneficial for CMPA patients is to distinguish allergies to baked or raw milk, thus determining prognosis and designing measures to induce milk tolerance [50].

The basophil activation test (BAT) is a promising, safe in vitro diagnostic method; although it is mostly used in research settings, it could provide a highly accurate diagnosis of IgE-mediated food allergies. During BAT, the activation of basophils via the IgE receptor leads to an increase in surface markers (CD63 and CD203c), whose level of expression is measured via the flow cytometry [24,38].

## 7. Treatment Options–Present and Future

The most widely accepted and utilized concept for the management of cow’s milk protein allergy is a “passive” approach, which means the complete elimination of cow’s milk and any dairy from the patient’s diet. While a high adherence to this diet leads to the resolution of symptoms in most cases, the risk of potentially life-threatening reactions and a possible detrimental impact on the nutritional status and overall quality of life of affected individuals warrant the continuous investigation of new, “proactive” therapeutic options. 

In the infant population, breastfeeding, if desired by the mother, can usually be continued if she is able to follow a strict dairy-free diet, thus eliminating the infant’s exposure to the allergen through breast milk. A switch to an extensively hydrolyzed formula (EHF) or, in the most severe cases of CMPA, amino-acid-based formula (AAF) is recommended [9,10]. Through hydrolyzation, IgE-binding epitopes “disappear”, thus preventing an allergic response [10]. Hydrolyzed peptides are proposed to exert active immunomodulatory effects: a strengthening of the epithelial barrier through the increase of regulatory cytokines (IL-10) and a decrease of inflammatory mediators (cyclooxygenase 2/COX-2/, NF-κB, IL-8) was observed in in vitro and ex vivo studies [14,51,52]. An increase in the number of Fox3+ Treg cells in the mesenteric lymph nodes was also noted in animal models with experimental colitis after the administration of casein hydrolysate [53,54].

In older children, a standard elimination diet and, in the case of involuntary allergen intake in severe IgE-mediated cases, carrying an adrenaline autoinjector are required to maintain remission and effectively mitigate life-threatening reactions; however, the focus is shifting toward therapeutic options that could possibly help to establish a state of sustained unresponsiveness or even permanent tolerance.

### Immunotherapy in CMPA Patients

Immunotherapy for food allergies can be classified based on the route the allergen is administered: oral immunotherapy (OIT) requires oral ingestion of the allergen; sublingual immunotherapy (SLIT) is characterized by holding the allergen under the tongue for 2 min; and epicutaneous immunotherapy (EPIT) relies on applying an allergen patch to the skin [29,55] (Table 3).

A conventional OIT protocol consists of three phases. During the initial escalation phase, which is carried out under supervision in a healthcare facility, the aim is to determine the starting dose (the highest safely tolerated dose) for daily administration at the patient’s home. This step is followed by a buildup phase, during which the daily dose is gradually increased at regular intervals (e.g., weekly or biweekly) until a maintenance dose is reached. Finally, this maintenance dose of the allergen should be consumed by the patient daily for an extended period (months or years). After the maintenance period, the daily ingestion is suspended for 4 to 12 weeks to assess sustained unresponsiveness with DBPCFC. A lack of a clinical reaction is indicative of a permanent tolerance [29,56].

The immunologic background of the mechanism of OIT remains unclear. It is proposed that frequent allergic stimulation leads to mast cell desensitization and the induction of allergen-specific Foxp3+ Tregs. Continuous high-dose allergen stimulation may induce IgG subclass switching (μ → γ3 → γ1 → γ2 → γ4) rather than sequential class switching (μ → γ3 → γ1 → ε, which would result in high-affinity IgE). Allergen-specific IgG4 antibodies are able to compete for allergen binding, thus inhibiting mast cell and basophil degranulation. IgG antibodies induced during OIT could act through the inhibitory receptor FcγIIb to decrease IgE-mediated hypersensitivity. In the early stages of OIT, dendritic cells produce IL-10, interferon- γ, and decreased levels of IL-6, which suppress the IgE-mediated hypersensitivity [17].

Combining OIT with omalizumab, an anti-IgE monoclonal antibody is studied as a possible augmentation of OIT efficacy through alleviating the severity of adverse reactions and facilitating a faster achievement of a maintenance dose [29,57,58]. A relatively low number of multicenter studies with this treatment regimen makes further investigation necessary [59,60,61].

SLIT and EPIT, although associated with fewer adverse reactions and better patient adherence, have scarcely been studied in patients with cow’s milk protein allergy; therefore, robust conclusions about their efficacy cannot be drawn [62,63,64]. According to previous studies, SLIT failed to produce the level of efficacy of OIT, although a multiple-fold increase in the tolerated allergen dose could still be achieved with milder, mainly localized adverse reactions [64]. A small pilot study for the assessment of the efficacy and safety of EPIT in patients with CMPA demonstrated a possibility for patients with severe reactions (e.g., anaphylaxis) to benefit from this therapeutic opportunity, after which they may tolerate OIT with fewer adverse effects [65].

Non-IgE-mediated CMPA has a more favorable prognosis, although the quickest possible achievement of oral tolerance is a reasonable objective in the therapeutic approach. Some studies suggest the beneficial role of probiotics (besides the elimination diet) in tolerance acquisition [66,67,68,69]. A deeper understanding of the pathophysiology of non-IgE-mediated CMPA may shed light on other possible therapeutic targets.

## 8. Conclusions

A continuous increase in the number of CMPA patients means a growing burden on the healthcare sector, children, and their families, which accentuates the importance of ongoing research in this field. A clearer insight into the pathomechanism of CMPA on a molecular level may facilitate the recognition of new potential therapeutic targets in the future.

While the molecular features of IgE-mediated allergies have been described in significant detail, some mechanisms of non-IgE-mediated allergies and mixed-background allergic diseases still remain enigmatic and need further research and understanding in order to explore a broader spectrum of diagnostic and therapeutic opportunities.

A paradigm shift (from “passive” to “proactive” management) is shaping the therapeutic approach to CMPA, which highlights the need for a thorough comprehension of the diverse immunologic mechanisms associated with cow’s milk ingestion in allergic individuals. Comprehensive knowledge about the mechanisms of the development of cow’s milk protein allergy can also further enhance current prevention strategies.

## Figures and Tables

**Table 1:** Most common symptoms of cow’s milk allergy, based on immunological background.

IgE Mediated Allergy (Type I)	Non-IgE Mediated Allergy (types III, IV)	Mixed Allergy (IgE- and Non-IgE- Mediated)
Urticaria Angioedema Abdominal pain/cramping Diarrhea (watery, with or without mucus) Vomiting Flushing Fainting Rhinitis Asthma Dyspnea Arrhythmia Atopic dermatitis Itchy, burning sensation	Gastrointestinal bleeding Protein-losing enteropathy Malabsorption Pulmonary disease Vasculitis Purpura Diarrhea Vomiting Anorexia Failure to thrive/weight loss Iron deficiency anemia Contact dermatitis	Dysphagia Abdominal pain Vomiting Diarrhea Malabsorption Weight loss Dysphagia Chronic reflux esophagitis Early satiety Delayed gastric emptying Gastric bleeding Sleep disturbance Anorexia

**Table 2:** Brief summary of the characteristics of main cow’s milk allergens (adapted from Linhart et al. [16]).

	Name	Molecular Weight	Structural and Functional Traits	Allergen Features
**Whey proteins**	Alpha-Lactalbumin (Bos d 4)	14.19 kDa	High amino acid sequence similarity to human counterpart Ca2+ binding protein with four stabilizing disulfide bridges	Genuine, milk-specific IgE epitopes clustered at the N- and C-terminal ends of the protein
	Beta-Lactalbumin (Bos d 5)	18.3 kDa	Lipocalin protein with two disulfide bridges (high stability to proteolytic cleavage) and one free cysteine	Linear IgE binding epitopes present in the amino acid sequence
	Serum albumin (Bos d 6)	67 kDa	High amino acid sequence similarity to human counterpart	Besides CMPA, it may play a role in beef allergy
	Immunoglobulin (Bos d 7)	160 kDa	4 polypeptide chains linked through intra- and intermolecular disulfide bonds	Allergenic activity uncertain; recognized by IgE in 10–40% of CMPA patients
	Lactoferrin (Bos d LF)	76.14 kDa	Iron-binding glycoprotein (antimicrobial effect through chelating iron and reducing bacterial iron uptake)	Unknown clinical relevance, recognized by IgE in 5–66% of CMPA patients
**Caseins**	AlphaS1-casein (Bos d 12) Alpha S2-casein (Bos d 10) Beta-casein (Bos d 11) Kappa-casein (Bos d 12)	22.89 kDa 24.35 kDa 23.58 kDa 18.97 kDa	Calcium-binding phosphoproteins Heat stable but highly susceptible to enzymatic degradation	Casein-specific IgE antibodies recognize linear epitopes

**Table 3:** Main features of different types of immunotherapy (modified from Barni S. et al. [29]).

	Oral Immunotherapy (OIT)	Sublingual Immunotherapy (SLIT)	Epicutaneous Immunotherapy (EPIT)
**Route of exposure**	Oral ingestion of allergen	Allergen placed under the tongue for 2 min	Patch with allergen placed on intact skin
**Adverse effects**	Major side effects (e.g., anaphylaxis) plausible, minor side effects (e.g., gastrointestinal) common	Minor side effects (localized, oropharyngeal reactions)	Minor side effects (e.g., localized skin irritation)
**Efficacy**	Higher desensitization rate	Moderate efficacy compared to OIT	Lack of data concerning CMPA, may be suitable for preparing high-risk patients for OIT
**Patient compliance**	May be moderate due to major side effects	Higher compliance compared with OIT	Higher compliance compared with OIT

## Data Availability

Data sharing not applicable.

## References

[1] Warren C.M., Jiang J., Gupta R.S. (2020). Epidemiology and Burden of Food Allergy. Curr. Allergy Asthma. Rep..

[2] Lendvai-Emmert D., Emmert V., Makai A., Fusz K., Prémusz V., Eklics K., Sarlós P., Tóth P. (2022). Fecal calprotectin levels in pediatric cow’s milk protein allergy. Front. Pediatr..

[3] Polgár M. (1997). Adverse Food Allergies [Adverz Táplálékreakciók.].

[4] Giannetti A., Vespasiani G.T., Ricci G., Miniaci A., di Palmo E. (2021). Cow’s Milk Protein Allergy as a Model of Food Allergies. Nutrients.

[5] Savage J., Johns C.B. (2015). Food allergy: Epidemiology and natural history. Immunol. Allergy Clin. N. Am..

[6] Labrosse R., Graham F., Caubet J.C. (2020). Non-IgE-Mediated Gastrointestinal Food Allergies in Children: An Update. Nutrients.

[7] Flom J.D., Sicherer S.H. (2019). Epidemiology of Cow’s Milk Allergy. Nutrients.

[8] Tordesillas L., Berin M.C., Sampson H.A. (2017). Immunology of Food Allergy. Immunity.

[9] Koletzko S., Niggemann B., Arato A., Dias J., Heuschkel R., Husby S., Mearin M., Papadopoulou A., Ruemmele F., Staiano A. (2012). Diagnostic approach and management of cow’s-milk protein allergy in infants and children: ESPGHAN GI Committee practical guidelines. J. Pediatr. Gastroenterol. Nutr..

[10] Arasi S., Cafarotti A., Fiocchi A. (2022). Cow’s milk allergy. Curr. Opin. Allergy Clin. Immunol..

[11] Caubet J.C., Ford L., Sickles L., Järvinen K., Sicherer S., Sampson H. (2014). Clinical features and resolution of food protein-induced enterocolitis syndrome: 10-year experience. J. Allergy Clin. Immunol..

[12] Zhang S., Sicherer S., Berin M. (2021). Pathophysiology of Non-IgE-Mediated Food Allergy. Immunotargets Ther..

[13] Gonsalves N. (2019). Eosinophilic Gastrointestinal Disorders. Clin. Rev. Allergy Immunol..

[14] D’Auria E., Salvatore S., Pozzi E., Mantegazza C., Sartorio M., Pensabene L., Baldassarre M., Agosti M., Vandenplas Y. (2019). Cow’s Milk Allergy: Immunomodulation by Dietary Intervention. Nutrients.

[15] Restani P., Ballabio C., Di Lorenzo C., Tripodi S. (2009). Molecular aspects of milk allergens and their role in clinical events. Anal. Bioanal. Chem..

[16] Linhart B., Freidl R., Elisyutina O., Khaitov M., Karaulov A. (2019). Molecular Approaches for Diagnosis, Therapy and Prevention of Cow’s Milk Allergy. Nutrients.

[17] Taniuchi S., Takahashi M., Soejima K., Hatano Y. (2017). Immunotherapy for cow’s milk allergy. Hum. Vaccin. Immunother..

[18] Nowak-Wegrzyn A., Bloom K., Sicherer S., Shreffler W., Noone S., Wanich N. (2008). Tolerance to extensively heated milk in children with cow’s milk allergy. J. Allergy Clin. Immunol..

[19] Shreffler W.G., Wanich N., Moloney M., Nowak-Wegrzyn A. (2009). Association of allergen-specific regulatory T cells with the onset of clinical tolerance to milk protein. J. Allergy Clin. Immunol..

[20] Bavaro S.L., De Angelis E., Barni S., Pilolli R., Mori F., Novembre E. (2019). Modulation of Milk Allergenicity by Baking Milk in Foods: A Proteomic Investigation. Nutrients.

[21] Tham E.H., Rajakulendran M., Van Bever H. (2020). Epicutaneous sensitization to food allergens in atopic dermatitis: What do we know?. Pediatr Allergy Immunol..

[22] Brough H.A., Nadeau K., Sindher S., Alkotob S., Chan S., Bahnson H., Leung D. (2020). Epicutaneous sensitization in the development of food allergy: What is the evidence and how can this be prevented?. Allergy.

[23] Hammerschmidt S.I., Friedrichsen M., Boelter J., Lyszkiewicz M., Kremmer E., Pabst O. (2011). Retinoic acid induces homing of protective T and B cells to the gut after subcutaneous immunization in mice. J. Clin. Investig..

[24] Yu W., Freeland D.M.H., Nadeau K.C. (2016). Food allergy: Immune mechanisms, diagnosis and immunotherapy. Nat. Rev. Immunol..

[25] McDole J.R., Wheeler L., McDonald K., Wang B., Konjufca V., Knoop K., Newberry R. (2012). Goblet cells deliver luminal antigen to CD103+ dendritic cells in the small intestine. Nature.

[26] Tordesillas L., Berin M.C. (2018). Mechanisms of Oral Tolerance. Clin. Rev. Allergy Immunol..

[27] Lee M., Lee Y., Song J., Lee J. (2018). Tissue-specific Role of CX(3)CR1 Expressing Immune Cells and Their Relationships with Human Disease. Immune Netw..

[28] Tan J., McKenzie C., Vuillermin P., Goverse G., Vinuesa C., Mebius R., Macia L. (2016). Dietary Fiber and Bacterial SCFA Enhance Oral Tolerance and Protect against Food Allergy through Diverse Cellular Pathways. Cell Rep..

[29] Barni S., Liccioli G., Sarti L., Giovannini M., Novembre E. (2020). Immunoglobulin E (IgE)-Mediated Food Allergy in Children: Epidemiology, Pathogenesis, Diagnosis, Prevention, and Management. Medicina.

[30] Kraus T.A., Brimnes J., Muong C., Liu J., Moran T., Tappenden K., Boros P. (2005). Induction of mucosal tolerance in Peyer’s patch-deficient, ligated small bowel loops. J. Clin. Investig..

[31] Spahn T.W., Weiner H., Rennert P., Lügering N., Fontana A., Domschke W. (2002). Mesenteric lymph nodes are critical for the induction of high-dose oral tolerance in the absence of Peyer’s patches. Eur. J. Immunol..

[32] Fujihashi K., Dohi T., Rennert P., Yamamoto M., Koga T., Kiyono H. (2001). Peyer’s patches are required for oral tolerance to proteins. Proc. Natl. Acad. Sci. USA.

[33] Dubois B., Joubert G., de Agüero M.G., Gouanvic M., Goubier A. (2009). Sequential role of plasmacytoid dendritic cells and regulatory T cells in oral tolerance. Gastroenterology.

[34] Goubier A., Dubois B., Gheit H., Joubert G., Villard-Truc F., Asselin-Paturel C., Trinchieri G. (2008). Plasmacytoid dendritic cells mediate oral tolerance. Immunity.

[35] Mucida D., Kutchukhidze N., Erazo A., Russo M., de Lafaille M.C. (2005). Oral tolerance in the absence of naturally occurring Tregs. J. Clin. Investig..

[36] Palomares O., Rückert B., Jartti T., Kücüksezer U., Puhakka T., Gomez E., Fahrner H., Speiser A., Jung A., Kwok W. (2012). Induction and maintenance of allergen-specific FOXP3+ Treg cells in human tonsils as potential first-line organs of oral tolerance. J. Allergy Clin. Immunol..

[37] Wambre E., Bajzik V., DeLong J., O’Brien K., Nguyen Q., Speake C., Gersuk V., DeBerg H., Whalen E., Ni C. (2017). A phenotypically and functionally distinct human T(H)2 cell subpopulation is associated with allergic disorders. Sci. Transl. Med..

[38] Ruinemans-Koerts J., Schmidt-Hieltjes Y., Jansen A., Savelkoul H., van Setten P. (2019). The Basophil Activation Test reduces the need for a food challenge test in children suspected of IgE-mediated cow’s milk allergy. Clin. Exp. Allergy.

[39] Lin X.P., Magnusson J., Ahlstedt S., Dahlman-Höglund A., Hanson L., Magnusson O., Bengtsson U. (2002). Local allergic reaction in food-hypersensitive adults despite a lack of systemic food-specific IgE. J. Allergy Clin. Immunol..

[40] Morita H., Nomura I., Orihara K., Yoshida K., Akasawa A., Tachimoto H., Ohtsuka Y., Namai Y., Futamura M., Shoda T. (2013). Antigen-specific T-cell responses in patients with non-IgE-mediated gastrointestinal food allergy are predominantly skewed to T(H)2. J. Allergy Clin. Immunol..

[41] Caubet J.C., Bencharitiwong R., Ross A., Sampson H., Berin M. (2017). Humoral and cellular responses to casein in patients with food protein-induced enterocolitis to cow’s milk. J. Allergy Clin. Immunol..

[42] Augustin M.T., Kokkonen J., Karttunen T.J. (2005). Duodenal cytotoxic lymphocytes in cow’s milk protein sensitive enteropathy and coeliac disease. Scand. J. Gastroenterol..

[43] Leung J., Beukema K.R., Shen A.H. (2015). Allergic mechanisms of Eosinophilic oesophagitis. Best Pract. Res. Clin. Gastroenterol..

[44] Jyonouchi S., Smith C., Saretta F., Abraham V., Ruymann K., Modayur-Chandramouleeswaran P., Wang M., Spergel J. (2014). Invariant natural killer T cells in children with eosinophilic esophagitis. Clin. Exp. Allergy.

[45] Murch S. (2006). Allergy and intestinal dysmotility—Evidence of genuine causal linkage?. Curr. Opin. Gastroenterol..

[46] Nakajima-Adachi H., Ebihara A., Kikuchi A., Ishida T., Sasaki K., Hirano K., Watanabe H., Asai K., Takahashi Y., Kanamori Y. (2006). Food antigen causes TH2-dependent enteropathy followed by tissue repair in T-cell receptor transgenic mice. J. Allergy Clin. Immunol..

[47] Schäppi M.G., Borrelli O., Knafelz D., Williams S., Smith V., Milla P. (2008). Mast cell-nerve interactions in children with functional dyspepsia. J. Pediatr. Gastroenterol. Nutr..

[48] Boyce J.A., Assa A., Burks A., Jones S., Sampson H., Wood R., Plaut M., Cooper S., Fenton M., Arshad S. (2010). Guidelines for the diagnosis and management of food allergy in the United States: Report of the NIAID-sponsored expert panel. J. Allergy Clin. Immunol..

[49] Tuano K.S., Davis C.M. (2015). Utility of Component-Resolved Diagnostics in Food Allergy. Curr. Allergy Asthma. Rep..

[50] Popielarz M., Krogulska A. (2021). The importance of component-resolved diagnostics in IgE-mediated cow’s milk allergy. Allergol. Immunopathol..

[51] Tanabe S. (2008). Analysis of food allergen structures and development of foods for allergic patients. Biosci. Biotechnol. Biochem..

[52] Visser J.T., Lammers K., Hoogendijk A., Boer M., Brugman S., Beijer-Liefers S., Zandvoort A., Harmsen H., Welling G., Stellaard F. (2010). Restoration of impaired intestinal barrier function by the hydrolysed casein diet contributes to the prevention of type 1 diabetes in the diabetes-prone BioBreeding rat. Diabetologia.

[53] Kiewiet M.B.G., Gros M., van Neerven R., de Vos P. (2015). Immunomodulating properties of protein hydrolysates for application in cow’s milk allergy. Pediatr. Allergy Immunol..

[54] Meulenbroek L.A., van Esch B., Hofman G., den Hartog Jager C., Nauta A., Willemsen L., Bruijnzeel-Koomen C., Garssen J., van Hoffen E. (2013). Oral treatment with β-lactoglobulin peptides prevents clinical symptoms in a mouse model for cow’s milk allergy. Pediatr. Allergy Immunol..

[55] van Esch B.C., Schouten B., de Kivit S., Hofman G., Knippels L., Willemsen L. (2011). Oral tolerance induction by partially hydrolyzed whey protein in mice is associated with enhanced numbers of Foxp3+ regulatory T-cells in the mesenteric lymph nodes. Pediatr. Allergy Immunol..

[56] Nurmatov U., Dhami S., Arasi S., Pajno G., Fernandez-Rivas M., Muraro A., Roberts G., Akdis C., Alvaro-Lozano M., Beyer K. (2017). Allergen immunotherapy for IgE-mediated food allergy: A systematic review and meta-analysis. Allergy.

[57] Gernez Y., Nowak-Węgrzyn A. (2017). Immunotherapy for Food Allergy: Are We There Yet?. J. Allergy Clin. Immunol. Pract..

[58] Wood R.A., Kim J.S., Lindblad R., Nadeau K., Henning A.K., Dawson P., Plaut M., Sampson H.A. (2016). A randomized, double-blind, placebo-controlled study of omalizumab combined with oral immunotherapy for the treatment of cow’s milk allergy. J. Allergy Clin. Immunol..

[59] Nadeau K.C., Schneider L., Hoyte L., Borras I. (2011). Rapid oral desensitization in combination with omalizumab therapy in patients with cow’s milk allergy. J. Allergy Clin. Immunol..

[60] Ibáñez-Sandín M.D., Escudero C., Morillo R.C., Lasa E., Marchán-Martín E., Sánchez-García S., Terrados S., Díaz C.G., Juste S., Martorell A. (2021). Oral immunotherapy in severe cow’s milk allergic patients treated with omalizumab: Real life survey from a Spanish registry. Pediatr. Allergy Immunol..

[61] Ayats-Vidal R., Riera-Rubió S., Valdesoiro-Navarrete L., García-González M., Larramona-Carrera H., Cruz O. (2022). Long-term outcome of omalizumab-assisted desensitisation to cow’s milk and eggs in patients refractory to conventional oral immunotherapy: Real-life study. Allergol. Immunopathol..

[62] Takahashi M., Soejima K., Taniuchi S., Hatano Y., Yamanouchi S., Ishikawa H., Irahara M., Sasaki Y., Kido H. (2017). Oral immunotherapy combined with omalizumab for high-risk cow’s milk allergy: A randomized controlled trial. Sci. Rep..

[63] Frischmeyer-Guerrerio P.A., Keet C., Guerrerio A., Chichester K., Bieneman A., Hamilton R., Wood R. (2014). Modulation of dendritic cell innate and adaptive immune functions by oral and sublingual immunotherapy. Clin. Immunol..

[64] de Boissieu D., Dupont C. (2006). Sublingual immunotherapy for cow’s milk protein allergy: A preliminary report. Allergy.

[65] Keet C.A., Frischmeyer-Guerrerio P.A., Thyagarajan A., Schroeder J.T., Hamilton R.G., Boden S., Steele P., Driggers S., Burks A.W., Wood R.A. (2012). The safety and efficacy of sublingual and oral immunotherapy for milk allergy. J. Allergy Clin. Immunol..

[66] Qamer S., Deshmukh M., Patole S. (2019). Probiotics for cow’s milk protein allergy: A systematic review of randomized controlled trials. Eur. J. Pediatr..

[67] Berni Canani R., Nocerino R., Terrin G., Coruzzo A., Cosenza L., Leone L. (2012). Effect of Lactobacillus GG on tolerance acquisition in infants with cow’s milk allergy: A randomized trial. J. Allergy Clin. Immunol..

[68] Cukrowska B., Ceregra A., Maciorkowska E., Surowska B., Zegadło-Mylik M., Konopka E., Trojanowska I., Zakrzewska M., Bierła J., Zakrzewski M. (2021). The Effectiveness of Probiotic Lactobacillus rhamnosus and Lactobacillus casei Strains in Children with Atopic Dermatitis and Cow’s Milk Protein Allergy: A Multicenter, Randomized, Double Blind, Placebo Controlled Study. Nutrients.

[69] Paparo L., Nocerino R., Bruno C., Di Scala C., Cosenza L., Bedogni G., Di Costanzo M., Mennini M., D’Argenio V., Salvatore F. (2019). Randomized controlled trial on the influence of dietary intervention on epigenetic mechanisms in children with cow’s milk allergy: The EPICMA study. Sci. Rep..

